# ID 320 - Adaptação Transcultural Brasileira de Declarações Informativas para Comunicar os Resultados de Revisões Sistemáticas de Intervenções Indicadas pelo Sistema GRADE

**DOI:** 10.5327/2237-9622.2025.v34s1.320

**Published:** 2025-11-25

**Authors:** Suena Medeiros Parahiba, Cintia Pereira de Araujo, Gilson Pires Dorneles, Airton Stein, Daniela Pachito, Haliton Alves de Oliveira, Juliana Carvalho Ferreira, Luís Claúdio Lemos Correia, Priscila Torres, Rachel Riera, Sarah Nascimento Silva, Tiago Farina Matos, Vania Cristina Canuto Santos, Verônica Colpani, Ávila Teixeira Vidal, Marta da Cunha Lobo Souto Maior, Cinara Stein, Maicon Falavigna

**Keywords:** certeza da evidência, síntese de evidências, desfechos em saúde

## Abstract

**Introdução:**

Uma comunicação transparente e de fácil compreensão pode melhorar a comunicação científica para informar os resultados de revisões sistemáticas (RS) aos tomadores de decisão em saúde. O grupo (GRADE) desenvolveu uma abordagem para aprimorar a comunicação dos resultados de RS de intervenções na língua inglesa. O objetivo deste estudo foi adaptar transculturalmente para a língua portuguesa do Brasil a abordagem para comunicar os resultados de RS indicada pelo grupo GRADE.

**Método:**

O processo de adaptação transcultural da abordagem para comunicar os resultados na língua portuguesa falada no Brasil foi realizado em seis etapas: 1) tradução da versão original por dois tradutores; 2) síntese das traduções; 3) retrotradução da versão consolidada; 4) avaliação por especialistas do GRADE; 5) pré-teste aplicando à versão validada pelos especialistas; 6) avaliação dos resultados quantitativos e qualitativos. A fase de pré-teste consistiu em um questionário estruturado para avaliação da concordância de pesquisadores brasileiros com as frases propostas para a conclusão de RS. A análise estatística foi realizada por meio de um teste de Gwet AC1 para avaliação da concordância dos respondentes com a frase proposta. O projeto foi aprovado pelo Comitê de Ética do Hospital Moinhos de Vento (5.771.941).

**Resultados:**

Este estudo adaptou 30 frases informativas para resultados de RS de intervenção de acordo com o grau de certeza da evidência proposto pelo método GRADE e os tamanhos de efeito grande, médio, pequeno mas importante, e nenhum efeito ou efeito trivial. Na etapa 4, um dos desafios foi ajustar os termos relacionados à classificação de baixa e moderada certeza da evidência: a tradução do termo em inglês, usado para baixa certeza da evidência, foi considerada pelos especialistas como moderada certeza. Como uma modificação mais significativa, entendeu-se que o uso do termo em português "moderado" para tamanho de efeito poderia causar confusão, pois também é utilizado para a classificação da certeza da evidência. Portanto, propôs-se o uso de "efeito médio" como alternativa a "efeito moderado". Na etapa 5, um total de 154 voluntários participou da fase de aplicação da versão validada pelos especialistas. Na etapa 6, o teste AC1 de Gwet revelou moderada a alta concordância (>60%) dos avaliadores com as frases propostas para resultados de RS com alta e moderada certeza da evidência, independentemente da faixa de efeito. Por outro lado, identificou-se baixa concordância dos avaliadores para as frases propostas nas faixas de efeito grande, médio, pequeno mas importante, e efeito trivial/nulo na certeza da evidência baixa.

**Conclusão:**

As principais contribuições foram relacionadas aos ajustes de termos que são distintos em inglês, mas que compartilham uma tradução semelhante em português quando realizada uma tradução literal. A concordância dos termos propostos possibilita a utilização no contexto brasileiro. Por meio de processos de adaptação cultural, é possível garantir um nível mais alto de entendimento e clareza, aumentando assim a eficácia de sua utilização pelos tomadores de decisão.

